# Development of a clinical prediction algorithm for knee osteoarthritis structural progression in a cohort study: value of adding measurement of subchondral bone density

**DOI:** 10.1186/s13075-017-1291-3

**Published:** 2017-05-16

**Authors:** Michael P. LaValley, Grace H. Lo, Lori Lyn Price, Jeffrey B. Driban, Charles B. Eaton, Timothy E. McAlindon

**Affiliations:** 1 Department of Biostatistics, Boston University School of Public Health, 801 Massachusetts Avenue 3rd Floor, Boston, MA 02118 USA; 2 Medical Care Line and Research Care Line, Houston VA HSR&D Center for Innovations in Quality, Effectiveness and Safety, Michael E. DeBakey Medical Center, Houston, TX 77030 USA; 3 Section of Immunology, Allergy, and Rheumatology, Baylor College of Medicine, 1 Baylor Plaza, BCM-285, Houston, TX 77030 USA; 4 Institute for Clinical Research and Health Policy Studies at Tufts Medical Center, Tufts Clinical and Translational Science Institute, Tufts University, 800 Washington Street, Box #63, Boston, MA 02111 USA; 5 Division of Rheumatology Tufts Medical Center, Box #406, 800 Washington Street, Boston, MA 02111 USA; 6 Department of Family Medicine, Alpert Medical School of Brown University, 111 Brewster Street, Pawtucket, RI 02860 USA

**Keywords:** Arthritis, Calibration, Discrimination, Joint space loss, Logistic regression, ROC curve, X-ray

## Abstract

**Background:**

Risk prediction algorithms increase understanding of which patients are at greatest risk of a harmful outcome. Our goal was to create a clinically useful prediction algorithm for structural progression of knee osteoarthritis (OA), using medial joint space loss as a proxy; and to quantify the benefit of including periarticular bone mineral density (BMD) in the algorithm.

**Methods:**

Participants were from the Osteoarthritis Initiative (OAI) Progression Cohort, with X-ray readings of medial joint space at 36- and 48-month visits, and a 30- or 36-month medial-to-lateral tibial BMD ratio (M:L BMD ratio) value. Loss of medial joint space was the outcome and clinically available factors associated with OA progression were employed in the base prediction algorithm, with M:L BMD ratio added to an enhanced prediction algorithm. The benefit of adding M:L BMD ratio was evaluated by change in area under the ROC curve (AUC), net reclassification improvement (NRI), and integrated discrimination improvement (IDI).

**Results:**

Five hundred thirty-three participants were included; 51 (14%) had medial joint space loss; 47% were female; the mean (SD) age was 64.6 (9.2) years and BMI was 29.6 (4.8) kg/m^2^. The base algorithm model included age, BMI, gender, recent injury, knee pain, and hand OA as predictors and had an AUC value of 0.65. The algorithm adding M:L BMD ratio had an AUC value of 0.73, and the AUC, NRI and IDI were all significantly improved (*p* ≤ 0.002).

**Conclusions:**

This clinical prediction algorithm predicts structural progression in individuals with OA using only clinically available predictors supplemented by the M:L BMD ratio, a biomarker that could be made available at clinical sites.

## Background

Osteoarthritis (OA) is the most common type of arthritis and is a substantial public health problem [1, 2]. Treatments for OA include nonsteroidal anti-inflammatory drugs (NSAIDs), physical therapy, and ultimately arthroplasty. A challenge in the clinical management of patients with OA is the variable pace of disease progression and prediction of which patients are in need of intervention [3]. Therefore, there is an important need for an algorithm that can stratify OA patients by their level of risk for progression over a short period of time. In addition, such an algorithm could be used to stratify subjects for inclusion in clinical trials for development of additional therapies [4].

There have been statistical models developed to predict the incidence [5–7] and progression [7–9] of structural changes in knee OA progression in the OA literature, however, their use may be difficult due to inclusion of radiographic or genetic biomarkers that require specialized equipment or personnel for assessment.

A different potential biomarker for knee OA structural progression is the medial-to-lateral tibial plateau bone mineral density ratio (M:L BMD ratio). This ratio can be acquired from dual-energy X-ray absorptiometry (DXA) scanners which are currently available for clinical imaging at many sites. The M:L BMD ratio may serve as a useful component of a prediction tool as it provides a measure of the bone underlying the knee, and the measurement can be semi-automated. DXA studies of periarticular bone have shown that knee compartments with localized OA have greater BMD than uninvolved regions [10–13]. This difference has been commonly standardized as the calculated ratio of BMD between the two compartments [11–17]. Use of this internal reference conveniently controls for individual variations in BMD [11] and the ratio is precise, with reported coefficients of variation ranging from 1 to 7% [10–16, 18, 19]. The M:L BMD ratio is predictive of knee OA severity [11, 13] and is highly correlated with compartment-specific joint space narrowing (JSN), osteophytes and sclerosis [16]. The co-location of radiographic sclerosis and region measured for the M:L BMD ratio suggests that these two measures may both reflect a similar process [16]. However, the M:L BMD ratio appears to be a more sensitive measure of this process because removal of radiographs which exhibit sclerosis on radiographs only partly attenuates its association with OA [16]. Furthermore, the M:L BMD ratio is associated with subchondral pathologies such as bone marrow lesions (which are themselves associated with OA progression) [15, 16, 20–25]. Two small longitudinal studies showed that unloading a knee is associated with a reduction of the M:L BMD ratio, suggesting that it is also responsive to changes in knee loading [11, 17].

### Study objectives

The first objective of this study was to develop and evaluate two prediction algorithms for knee OA structural progression in participants with existing knee OA. These algorithms will be (1) a base model, which includes factors that are currently readily available in a typical setting, and (2) an enhanced model that adds the M:L BMD ratio to the base model. The second objective was to quantify the improvement in prediction obtained when the M:L BMD ratio is added to the base model.

## Methods

### Source of study participants: the Osteoarthritis Initiative (OAI) progression cohort

The OAI is a multicenter cohort study that collected longitudinal clinical and image data in participants with, or at high risk of, knee OA over a 9-year period [26]. OAI progression cohort participants were 45–79 years of age at recruitment in the clinical sites of Baltimore, MD, Pawtucket, RI, Columbus, OH and Pittsburgh, PA, and had symptomatic knee OA in at least one knee.

So that the study sample mirrored subjects in clinical care for knee OA, we included participants from the progression cohort of the OAI. At the OAI baseline visit, these participants had knee pain, aching or stiffness on most days of 1 month of the preceding year and radiographic tibiofemoral knee OA (Osteoarthritis Research Society International (OARSI) atlas grades 1–3) on a fixed-flexion radiograph at recruitment in at least one knee, but not necessarily in their study knee. Most subjects had radiographic OA in their study knee, but we also chose to include the participants without OA in their study knee in our analyses to reflect clinical care of patients with contralateral OA. More information on the inclusion and exclusion criteria for the OAI progression cohort is available at http://oai.epi-ucsf.org. This study was approved by the Institutional Review Boards at the four clinical sites, Baylor College of Medicine, and Tufts Medical Center.

The goal of the Bone Ancillary Study was to investigate the influence of bone on structural progression in knee OA. Participants in this ancillary study were recruited from the four OAI clinical sites at the 30- or 36-month visit at which visit knee DXA scans were acquired (August 2007 to April 2009). While 600 participants was the ancillary study enrollment target for the progression cohort, 629 were ultimately enrolled. For this analysis we used progression cohort participants who were included in the Bone Ancillary Study who also had radiographic readings from the 36- and 48-month OAI examinations.

### Outcome: progression measures

At the 24-, 36- and 48-month examinations, posterior-anterior radiographs were taken with a fixed flexion in weight-bearing position using a positioning frame to provide optimal assessment of change in joint space. All radiographs were read for medial and lateral joint space using the OARSI 0–3 scale [27] by trained readers and within grade scores (e.g., 2.2, 2.4, etc.) were allowed. The reliability for these readings (read-reread) was high (weighted kappa [intra-rater reliability] = 0.88 [95% CI 0.80–0.95]). The 24-month radiographs were further evaluated for the Kellgren and Lawrence grade [28], and we use the knees with grades of 2 or more in subset analyses of knees with radiographic OA. All radiographic readings were obtained from the OAI public data release (kXR_SQ_BU_03 (versions 5.5 and 6.3) and kXR_SQ_BU_06).

Our measurement for structural OA progression was loss of medial joint space within a single knee from each participant on the radiograph between the 36- and 48-month examinations. We did not include loss of lateral joint space as a progression as there were few of these and they may reflect a different process. We took any increase in the medial joint space value, including increases within an OARSI grade (such as from 2.0 to 2.4), as a progression event. In addition, any knee replacements that occurred by the 48-month visit in study knees with predominantly medial involvement at the 36-month radiograph, by consensus of the authors, were counted as progression events.

### Exposure (biomarker): M:L BMD ratio

Our exposure of main interest is the M:L BMD ratio, a DXA imaging biomarker for knee OA [29]. The M:L BMD ratio measure of the study knee was acquired at recruitment of a participant into the Bone Ancillary Study, either at the OAI 30- or 36-month examination, using the techniques described below.

### Tibial plateau dual-energy X-ray absorptiometry

**Fig. 1:**
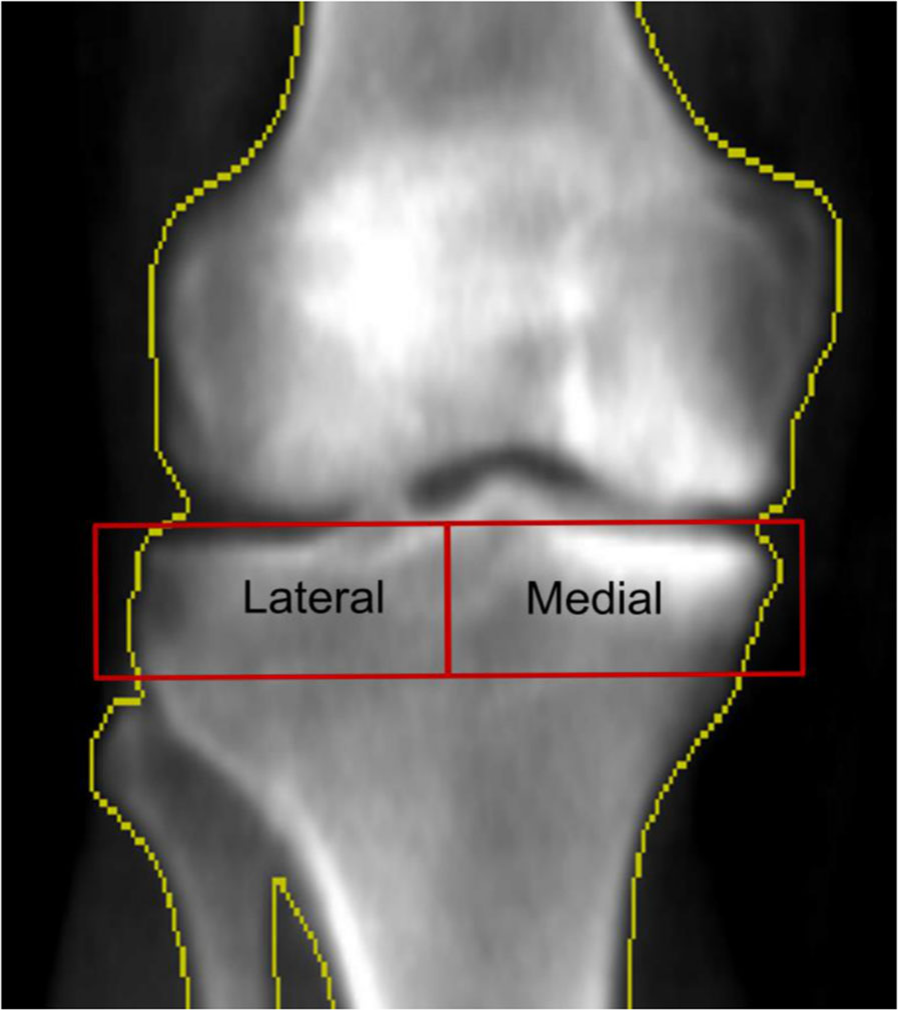
DXA knee scan with regions of interest for the M:L BMD ratio

The proximal tibiae were evaluated using one of four identical DXA systems (Lunar Prodigy Advance, GE Lunar Corp., Madison WI, USA) at each OAI clinical site with investigational knee software (enCORE 2007 Version 11.20.068). The lower extremity was positioned with the long axis of the tibia perpendicular to the X-ray beam, and neutrally rotated. A foam knee positioner, which was posterior to the popliteal fossa, placed the knee in mild flexion. The foot positioner was then applied to the ipsilateral foot using a Velcro strap around the perimeter of the foot and the positioner. This resulted in the ipsilateral toes oriented perpendicular to the scanning bed and the plantar surface of the ipsilateral foot adjacent to the perpendicular edge of the positioner, parallel to the lower border of the scanner bed. A positioning laser centered the scanner arm 5 centimeters below the inferior pole of the patella. The knee was then imaged using DXA (Fig. 1).

### Tibial plateau dual-energy X-ray absorptiometry image analysis

Two DXA analysts determined the tibial plateau BMD measurements using a customizable region of interest. The height of the medial and lateral tibial regions of interest was fixed at 20 mm while the region of interest width in the medio-lateral direction was set as half the distance between the medial and lateral bone edges along a line midway between the far medial and lateral points of the tibial plateau (Fig. 1) [14]. The bone edges (*yellow outline*) served as the outer border of the region of interests. The regions of interest were positioned so that their top edges were just superior and parallel to the medial joint surfaces of the tibia [14]. The medial and lateral tibial bone mineral densities were divided to generate M:L BMD ratio scores. The scan-rescan (with repositioning) intra-class correlation was 0.993 (95% CI 0.982–0.997) [29]. Both the intra-rater reliability and inter-rater reliability (measurement-remeasurement) were excellent at 0.98–0.99.

### Predictive factors

For our base model we evaluated readily available clinical predictors from the OAI data, based on their reported connection to knee OA structural progression in the literature, with the consideration that there should be close to ten progression events per included predictor [30]. From the literature we chose to include age, gender and body mass index (BMI), knee pain and injury regardless of statistical significance [31, 32] Other potential predictors were screened in bivariate analyses with progression in our study sample, and included if their *p* values were 0.2 or less. While radiographic measures, such as Kellgren and Lawrence grade, joint space width, or knee alignment, can be strong predictors of OA progression, we did not include these in the prediction models as they would require advanced radiographic assessment which is not standard in routine clinical management for OA. However, we did include the presence of Kellgren and Lawrence grade of 2 or more at the 24-month examination as a descriptive factor and to perform a subgroup analysis restricted to knees with radiographic OA.

In our base model we included gender, age, BMI, knee pain, previous injury to the knee (yes/no), and the presence of hand OA (yes/no). BMI was calculated by dividing the weight of the subject in kilograms by the height (squared) of the subject in meters, with both measures taken from the DXA examination evaluation. The knee pain assessment was from a 0–10 visual analog scale for pain in the study knee from the 36-month examination. The presence of a knee injury was taken to be a positive response to the question “Were you injured badly enough to limit ability to walk for at least 2 days, since last visit about 12 months ago?” administered at the 36-month examination. We judged hand OA to be present if three or more Heberden’s nodes (from both hands) were detected in the clinical examination at recruitment to the OAI as this cutoff gave the strongest association with progression. Predictive factors and presence of radiographic OA were compared between knees which were excluded from analysis due to lack of a measure of 48-month medial joint space and those included in the prediction analysis. Logistic regression models were used to provide univariate and multivariable adjusted odds ratios for association with progression.

### Statistical modeling and assessment

To evaluate the linearity of association between continuous predictors and the dichotomous outcome of OA progression (yes/no), we compared the goodness of fit of logistic regression models using either natural cubic splines that can accommodate non-linear effects or linear trends to model the association [33, 34]. The base prediction model was constructed using logistic regression and the predictive factors listed above. The BMD enhanced prediction model was constructed using logistic regression with the same predictive factors but also adding the M:L BMD ratio biomarker. The analysis used the presence of loss in medial joint space between the 36- and 48-month examinations as the outcome as the biomarker assessment strictly precedes all joint space measures used for the outcome. In a subgroup analysis, we performed the same evaluation restricted to subjects whose knees had more advanced osteoarthritis as measured by a Kellgren and Lawrence score of 2 or more at the 24-month examination.

The prediction models were evaluated on two characteristics – discrimination and calibration. Discrimination measures how well the model distinguishes between participants who experience OA progression and those who do not. Discrimination was assessed using area under the curve (AUC), with guidelines suggesting that values of at least 0.70 are needed for adequate prediction [35]. Calibration measures if the model systematically over or under-predicts OA progression. While calibration is often assessed using the Hosmer and Lemeshow test, this approach has been criticized [36] and we employ an approach which evaluates the intercept and slope from a new logistic regression model fit to the same outcome and using only the predictions from the preceding model as a predictor. If the new logistic regression intercept is close to 0 and the slope close to 1 then the initial model is well calibrated.

To evaluate whether a new biomarker is informative beyond previously established markers and predictors, the predictive ability of the model enhanced with the new biomarker is compared to the base model. Change in the AUC between models gives the change in overall discrimination; the net reclassification improvement (NRI) adds the percent of observations with OA progression that have an increased predicted value in the enhanced model and the percent of observations without progression that have a reduction in predicted value. The integrated discrimination improvement (IDI) adds the average increase in predicted values in the observations with OA progression to the average decrease in predicted values in observations without OA progression [37]. As a prediction algorithm based on a logistic regression model with few OA progression events per predictor is prone to produce overly optimistic estimates that do not generalize to other data sets, so we used an internal validation approach to reduce this bias [33]. The internal validation uses a tenfold cross-validation procedure to produce predicted values with reduced bias for use in assessing the logistic regression discrimination, calibration, and improvement measures.

## Results

### Description of cohort

Out of 629 Bone Ancillary Study participants from the OAI progression subcohort, 96 participants were excluded due to indeterminate medial progression values (missing the 48-month visit, or unreadable medial joint space on either the 36 or 48-month radiograph), leaving 533 participants for the primary analysis. The excluded subjects had a higher mean BMI (*p* = 0.04) and had higher percentages of females (*p* < 0.01), knee injury (*p* < 0.01), and radiographic OA (*p* < 0.01) than subjects remaining in the analysis.

There were 51 knees with medial joint space progression (9.6%), including five knees which underwent total knee replacement with predominately medial OA at 36 months. Table 1 provides the descriptive characterization of these knees both overall and by progression status.

**Table 1:** Descriptive characteristics by primary analysis outcome

Characteristic	Overall (*n* = 533)	Non-progressors (*n* = 482)	Progressors (*n* = 51)
Female (*n*, %)	252 (47%)	226 (47%)	26 (51%)
Age years (mean, SD)	64.6 (9.2)	64.2 (9.2)	68.3 (7.5)
Body mass index kg/m^2^ (mean, SD)	29.6 (4.8)	29.5 (4.7)	30.4 (4.9)
Knee pain (mean, SD)	3.5 (2.8)	3.4 (2.8)	4.1 (2.7)
Recent injury (*n*, %)	11 (2%)	9 (2%)	2 (4%)
Hand OA (*n*, %)	191 (36%)	160 (33%)	31 (61%)
M:L BMD (mean, SD)	1.1 (0.2)	1.1 (0.1)	1.2 (0.2)
Radiographic knee OA (*n*, %)	369 (69%)	324 (67%)	45 (88%)

*OA* osteoarthritis, *M:L BMD ratio* medial-to-lateral tibial plateau bone mineral density ratio

### Logistic regression modeling

Univariate and multivariate odds ratios for the associations between descriptive characteristics and OA progression are shown in Table 2. The continuous values of age, body mass index, and M:L BMD are modeled as linear terms in the regression corresponding to a one standard deviation increase in value.

**Table 2:** Univariate and multivariate associations with the primary analysis outcome

Characteristic	Univariate odds ratio (95% CI)	Multivariable odds ratio (95% CI)
Female	1.18 (0.66, 2.11)	1.06 (0.56, 2.01)
Age, years^a^	1.58 (1.18, 2.16)	1.45 (1.02, 2.08)
Body mass index kg/m^2 a^	1.20 (0.90, 1.59)	1.32 (0.95, 1.84)
Knee pain^a^	1.28 (0.96, 1.70)	1.06 (0.77, 1.47)
Recent injury	2.14 (0.32, 8.60)	2.58 (0.39, 16.92)
Hand OA	3.12 (1.74, 5.72)	2.68 (1.39, 5.19)
M:L BMD^a^	1.94 (1.47, 2.58)	1.76 (1.32, 2.35)
Radiographic knee OA	3.33 (1.50, 8.87)	2.20 (0.88, 5.50)

*OA* osteoarthritis, *M:L BMD ratio* medial-to-lateral tibial plateau bone mineral density ratio

^a^Odds ratio estimates per 1 standard deviation change in predictor

**Fig. 2:**
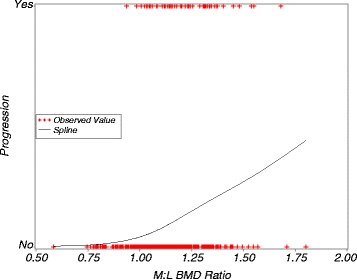
Unadjusted predicted risk of medial progression from univariate logistic regression with a natural cubic spline for M:L BMD ratio. Uses the outcomes from the primary analysis. *M:L BMD ratio* medial-to-lateral tibial plateau bone mineral density ratio

Initial analyses indicated that the M:L BMD ratio, age and BMI had non-linear trends as predictors of medial joint space loss, and were modeled using natural cubic splines. The knee pain measure did show a linear trend with medial joint space loss and was used as a linear predictor. Figure 2 shows the form of the spline for M:L BMD ratio in the initial analysis: the risk of medial joint space loss is close to 0 for ratio values below 1, there is an upward bend for values near 1, and then a linear increase in the risk for ratio values above 1.

The base model includes age and BMI as natural cubic splines, knee pain as a linear term, and sex, recent injury, and presence of hand OA as dichotomous predictors. The BMD enhanced model has the same set of predictors plus a natural cubic spline for the M:L BMD ratio. Adding in the spline for M:L BMD increases the AUC measure of discrimination from 0.65 in the base model to 0.73 in the BMD enhanced model, for a difference of 0.08 with a 95% confidence interval from 0.03 to 0.13 (*p* = 0.002). The BMD enhanced model has an AUC value above the threshold of 0.70 which denotes acceptable discrimination [35]. Both models show good calibration with intercept estimates equal to 0 out to 5 decimal places, and slopes equal to 1 out to 4 decimal places.

### Evaluation of improvement in prediction with M:L BMD

**Fig. 3:**
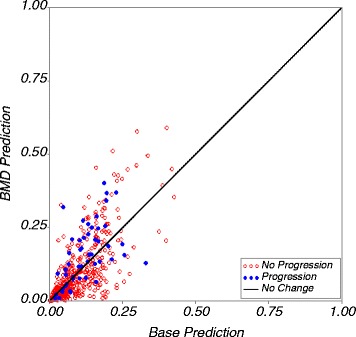
Predicted risk of progression from the model including M:L BMD ratio on the Y-axis plotted against predicted probability of progression in the base model on the X-axis for the primary analysis outcome. A strong biomarker is shown by having the participants with progression *above the diagonal line* and the participants without progression *below the line. BMD* bone mineral density

Figure 3 shows the cross-validated predicted probability of progression from the base model on the horizontal axis and the cross-validated predicted probability from the BMD enhanced model on the vertical axis for each participant. Improvement in prediction in the BMD enhanced model compared to the base model is shown by most participants with OA progression (*solid blue points*) appearing above the diagonal line and most participants without progression (*red circles*) below the diagonal. The results displayed in Fig. 3 are quantified using the NRI and IDI in Table 3. The percentage of the 51 subjects who did experience progression who had an increased predicted risk or progression from the BMD enhanced model compared to the base model minus those with a decrease in risk was 22%. In the 482 subjects without progression, the net percentage of subjects with decreased predicted risk was 27%. From these values, the overall NRI value is 0.49 and this is statistically significant (*p* = 0.001). In participants with joint space loss there is an average increase in predicted value of 0.04 in the BMD enhanced model compared to the base; and the average predicted value is the same in both models for participants without joint space loss. This gives an IDI value of 0.04 which is also statistically significant (*p* = 0.002).

**Table 3:** Improvement measures comparing base and BMD models in primary analysis

Improvement measure	Outcome	Base model	BMD model	Comparison
Net reclassification improvement			Net % improvement	NRI (95% CI)
	OA progression	Ref	22%	0.49 (0.20, 0.77)
	No OA progression	Ref	27%	
Integrated discrimination improvement		Base model average prediction	BMD model average prediction	IDI (95% CI)
	OA progression	0.12	0.16	0.04 (0.01, 0.06)
	No OA progression	0.09	0.09	

*BMD* bone mineral density, *OA* osteoarthritis

In the subgroup analysis that was restricted to the 365 knees with Kellgren and Lawrence grade of 2 or more at the 24-month examination, 45 had medial joint space progression (12.3%). While the AUC, NRI and IDI values were as good or better than those in the full analysis, neither the base nor BMD enhanced model had adequate calibration. The intercept estimates were far from 0 (-0.99 in the base model, and -0.63 for the BMD enhanced model), and the slopes well below 1 (0.47 in the base model, 0.63 in the BMD enhanced model). The slopes below 1 indicate that the predictions were too extreme in these subgroup models – low predictions were exaggeratedly low, and high predictions exaggeratedly high – indicating the subgroup was too small to adequately fit the models [36].

## Discussion

We found the M:L BMD ratio together with routine clinically available patient characteristics to provide acceptable prediction of loss of medial joint space over 1 year of follow-up. In addition, the M:L BMD ratio provided a significant improvement in prediction above what would be possible using only the clinically available patient characteristics, using both the net reclassification improvement and the integrated discrimination improvement measures. However, when restricted to the subgroup of knees with advanced OA (Kellgren and Lawrence grade of 2 or more), the M:L BMD ratio model was not well-calibrated, which could indicate the model having too many terms to estimate with the reduced sample size.

There have been five sets of prediction rules developed for knee OA of which we are aware: Zhang et al. used clinical factors along with radiographic information to develop two prediction rules for incidence and one for progression of knee OA [7]; Kraus et al. used clinical data along with a radiographic fractal signature analysis to develop a prediction rule for progressive OA [9]; Kerkhof et al. used clinical, radiographic and genetic information to predict incidence of knee OA [5]; Blanco et al. used clinical and genetic data to predict progression to end-stage OA (Kellgren and Lawrence grade 4 or total knee replacement) [8]; and Losina et al. used their osteoarthritis policy model to calculate lifetime risk of symptomatic knee OA using demographic, occupational, family history, and medical diagnosis of knee OA [6]. While we focused on progression of knee OA, our prediction model is different than those of Zhang, Kraus, Kerkhof, Blanco, or Losina. To maintain clinical utility for our prediction model, only easily obtainable patient and clinical factors were used except for the M:L BMD ratio biomarker, which would provide a new usage of the broadly available DXA scanners. In addition, previous efforts have based outcomes directly on changes in Kellgren and Lawrence grade, where we have focused on the medial compartment to reduce heterogeneity in the outcome.

### Limitations of study

Current literature on prediction rules call for validation of rules on external datasets, as the results are generally less impressive in data not used to generate the prediction rules. However, we did not have access to another dataset with both measures of OA progression and the M:L BMD ratio biomarker. Our use of cross-validation did reduce the bias in assessment of the prediction algorithms, but ideally these should be evaluated in an independent dataset. We hope to perform this external validation in future work. In addition, even though we are using a large number of participants with symptomatic knee OA, the number of progression events was not as large as would be ideal for development of a prediction rule. This led us to be more sparing in the use of potential predictors in the base model than we would have preferred.

As our interest was to create prediction rules for OA progression that would be relevant for patients in clinical care for osteoarthritis we used subjects with initial findings of osteoarthritis from the OAI cohort. However, some knees still had OARSI grade 0, i.e., intact medial joint space, at the 36-month evaluation. So, our progression measure includes what might be considered incident joint space loss as well as progression of previously reduced joint space. As incident and progressive OA may have different risk factors, our progression model may mix these disparate predictors. However, these are still the predictors that would apply to patients in clinical care with a mix of incident and progressive disease at different locations with the knee. In addition, we have attempted to use the predictors that would be available in the clinical context, with the exception of our main exposure, the M:L BMD value. While this is not available in clinical care, it could provide a means of using widely available DXA scanners to better utilize the quality of bone in caring for OA patients. In addition, we have limited our evaluation to changes in the medial joint space, which excludes the less common, but still important, lateral compartment changes.

Finally, we note that there has been controversy over how to best study the progression of OA as it is challenging to provide causal interpretations for adjusted estimates in studies of subjects with established disease [38]. However, these predictive algorithms and models do not address the causality of exposures and biomarkers, just their ability to provide an early warning of future disease outcomes. So, these concerns regarding causality do not apply to this research.

## Conclusions

So, in conclusion, we found that a prediction model composed of patient characteristics and the M:L BMD ratio provided adequate prediction of medial-compartment knee OA progression. The M:L ratio biomarker provided an important component of this prediction rule as it statistically significantly increased the discrimination and enhanced the predictive ability of this rule.

## Abbreviations

AUC: Area under the curve
BMD: Bone mineral density
BMI: Body mass index
DXA: Dual-energy X-ray absorptiometry
IDI: Integrated discrimination improvement
JSN: Joint space narrowing
M:L BMD ratio: Medial-to-lateral tibial plateau bone mineral density ratio
NRI: Net reclassification improvement
OA: Osteoarthritis
OAI: Osteoarthritis Initiative
OARSI: Osteoarthritis Research Society International
